# Single Event Upset Study of 22 nm Fully Depleted Silicon-on-Insulator Static Random Access Memory with Charge Sharing Effect

**DOI:** 10.3390/mi14081620

**Published:** 2023-08-17

**Authors:** Chenyu Yin, Tianzhi Gao, Hao Wei, Yaolin Chen, Hongxia Liu

**Affiliations:** Key Laboratory for Wide Band Gap Semiconductor Materials and Devices of Education Ministry, School of Microelectronics, Xidian University, Xi’an 710071, China; yin_chenyu@163.com (C.Y.);

**Keywords:** fully depleted silicon-on-insulator (FDSOI), single event upset (SEU), SRAM, charge sharing effect, 3D device simulation, bipolar amplification effect

## Abstract

In this paper, the single event effect of 6T-SRAM is simulated at circuit level and device level based on a 22 nm fully depleted silicon-on-insulator (FDSOI) process, and the effects of charge sharing and bipolar amplification are considered in device-level simulation. The results demonstrate that, under the combined influence of these two effects, the circuit’s upset threshold and critical charge decreased by 15.4% and 23.5%, respectively. This indicates that the charge sharing effect exacerbates the single event effects. By analyzing the incident conditions of two different incident radius particles, it is concluded that the particles with a smaller incident radius have a worse impact on the SRAM circuit, and are more likely to cause the single event upset in the circuit, indicating that the ionization distribution generated by the incident particle affects the charge collection.

## 1. Introduction

As a storage circuit, static random access memory (SRAM) has been widely used in the field of integrated circuits due to its advantages of fast speed and stable operation. Its reliability has always been the focus of researchers. In the space environment, a SRAM circuit is subjected to a single event effect (SEE) due to the incident of high energy particles. As a common soft error, single event upset (SEU) causes the node potential of SRAM to flip, destroying the original storage logic and affecting the normal operation of the circuit [1]. To improve the single event resistance of SRAM, researchers have proposed various reinforcement schemes. A fully depleted silicon-on-insulator (FDSOI) has an ultra-thin top layer of silicon and a buried oxide layer (BOX) composed of silicon dioxide, which can effectively isolate the collection of charges by the electrode and improve the anti-single event capability of the device. Experiments show [2] that FDSOIs have low SEE sensitivity and a high total ionizing dose (TID) resistance, which is very suitable for applications in the space environment.

With the increasing integration of circuits, the number of transistors per unit area on the wafer is increasing, and the distance between devices is shrinking, aggravating the charge sharing effect [3]. Studies have shown that although FDSOI devices provide better resistance to SEU than comparable bulk technologies, there is also a charge sharing effect in the circuit which will lead to single event multi-bit upset (SEMU) in the circuit [4]. The most direct way to study the SEE of SRAM is to verify the circuit through satellite in orbit or ground test experiments, but the experimental verification is not only expensive, but also the impact on the SEE cannot be observed the first time. Therefore, it is particularly important to establish accurate simulation models for the study of SEE.

**Figure 1:**
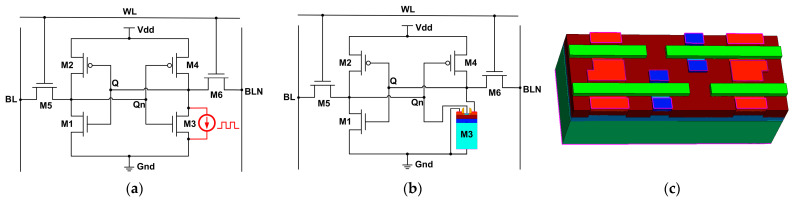
Simulation method of SRAM single event effect: (**a**) circuit level; (**b**) mixed level; (**c**) device level.

As shown in Figure 1, the SEU simulation research of SRAM can be divided into device level, mixed level and circuit level, according to the research methods. Each of the three research methods has its own advantages and disadvantages [5,6]. The circuit-level simulation simulates the single event transient (SET) current generated by adding a pulsed current source model to the sensitive nodes to obtain the voltage variation at each node of the circuit during single-particle incidence [7]. The advantage of this approach is its fast simulation speed and simplicity. However, it neglects the impact of the changing electrode voltages of the irradiated device on the bipolar amplification effect, which in turn affects the magnitude of the SET current. The electrode voltage of the incident device affects the magnitude of the SET current. The incident of single particle makes the electrode voltage of the device change dynamically, which leads to a dynamic change in the SET current. Therefore, it is deficient to describe the single event transient pulse current only by a fixed pulse current source. Based on the circuit level simulation, the mixed level simulation replaces the model of the incident device with the device level model, and the remaining devices adopt the circuit level model [8]. This method takes into account the influence of the device electrode voltage on the SET current while ensuring the speed of circuit simulation. Compared with the circuit level simulation, the results are more realistic. However, since only the incident devices in the circuit are modeled at the device level and the devices are connected to each other by electrodes, the charge sharing effect and SEMU cannot be described. The device level simulation is achieved by unified device level modeling of the SRAM circuit as a whole [9]. Among the three methods, device level simulation provides the most accurate and realistic results. However, device level simulation is the slowest and requires a lot of time, and complex models may lead to non-convergence of the simulation.

In this paper, an SEU simulation at SRAM circuit level and device level is implemented by Hspice and Synopsys technology computer aided design (TCAD) Sentaurus simulator. The results of the two methods are compared to analyze the effect of charge sharing on the flip threshold of a 22 nm FDSOI SRAM cell. This paper can improve the accuracy of SEU prediction and lay a foundation for the SRAM reinforcement design of advanced technology.

## 2. Device Structure

**Figure 2:**
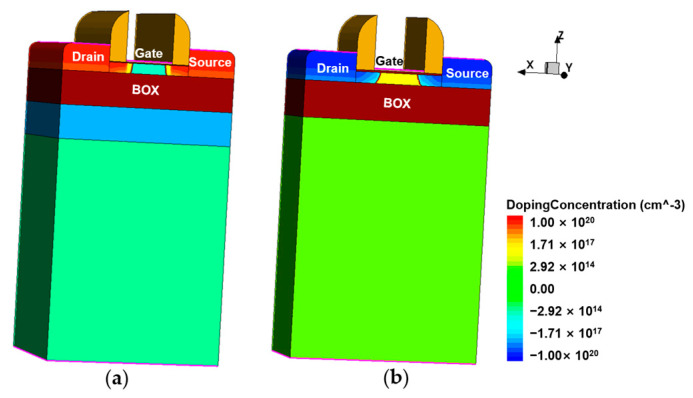
22 nm FDSOI device structure: (**a**) NFET; (**b**) PFET.

The NMOS (N-type Metal-Oxide-Semiconductor), Field Effect Transistor (NFET), and PMOS (P-type Metal-Oxide-Semiconductor) Field Effect Transistor (PFET) with FDSOI structure are built by Synopsys TCAD Sentaurus simulator based on the layout size parameters of process design kits (PDK) in 22 nm FDSOI process provided by Global Foundries, and Figure 2 shows the three-dimensional (3D) structure of the devices. The source and drain regions of the device adopt Gaussian doping distribution, and HfO_2_ with high dielectric constant is added to thin silicon dioxide (SiO_2_) gate oxide to reduce gate leakage current and improve channel mobility. More detailed parameters are listed in Table 1.

**Figure 3:**
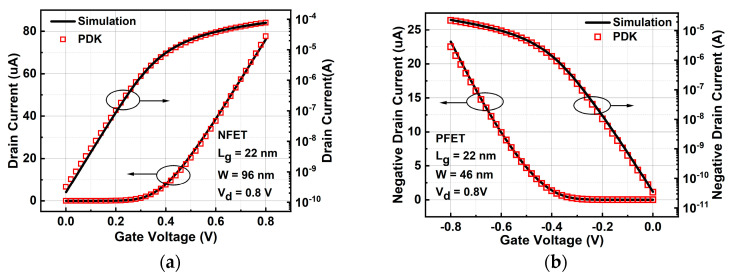
Comparison of transfer characteristics of drain current for (**a**) NFET and (**b**) PFET with the PDK data.

The transfer characteristic curve of PDK is obtained by calling the 22 nm FDSOI process library using Hspice (Version R-2020.12) simulation software to calibrate the device model. The calibration results are shown in Figure 3, and the direct current (DC) parameters extracted from the curve are shown in Table 2.

**Figure 4:**
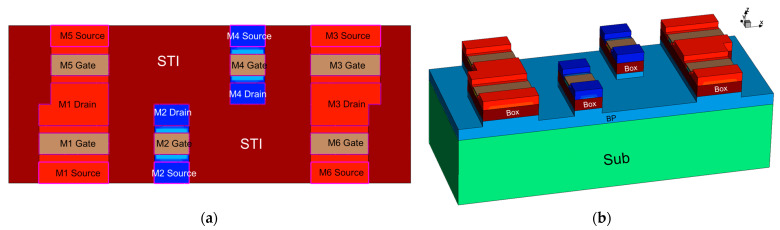
6T-SRAM device model structure. (**a**) vertical view. (**b**) oblique view.

**Figure 5:**
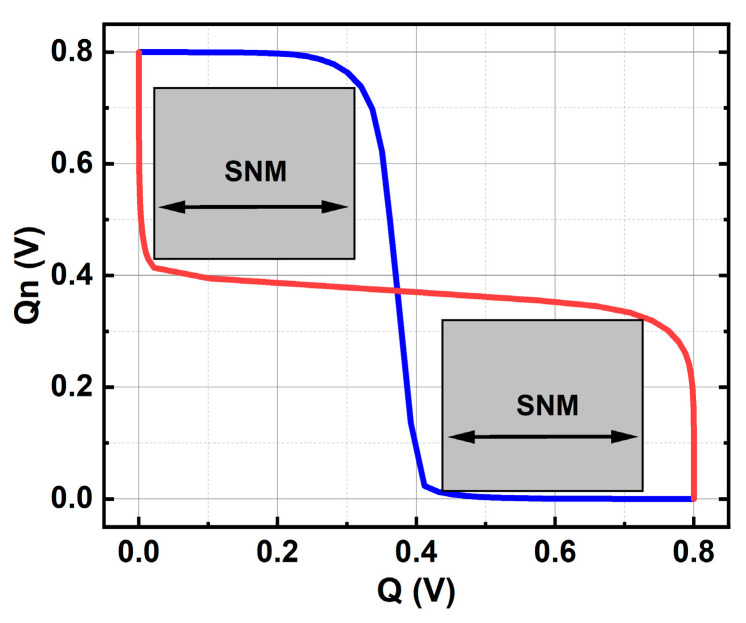
The butterfly Curve of 6T-SRAM.

According to the layout structure of 6T-SRAM in PDK, the 6T-SRAM device model is established in 1:1 ratio. The 6T-SRAM device model uses the same doping settings as the FDSOI NFET and PFET. As shown in Figure 4, in an SRAM layout, adjacent NEFTs merge their active regions based on the circuit’s connectivity, forming a source-drain sharing structure [10]. This is a key distinction between the single-device model and the multi-device model. The read and write simulation of SRAM is carried out, and the noise tolerance of SRAM is further obtained as shown in Figure 5. By simulating the read and write of SRAM, the noise tolerance of SRAM is shown in Figure 5.

## 3. SRAM Circuit-Level SEE Simulation

### 3.1. SET Current Extraction

For FDSOI devices, the direction, angle, energy, and ambient temperature of the single particle incident have an effect on the SET current. In the study of SET, a linear energy transfer (*LET*) is introduced to describe the energy lost per unit length of the particle passing through the target material. The expression is shown in Equation (1):

(1) $$LET=\frac{1}{\rho }\frac{dE}{dx}$$

where ρ is the density of the semiconductor material, E is the energy of the incident particle, and x is the trajectory distance generated by the incident particle. The common units of *LET* are $\mathrm{MeV}\cdot {\mathrm{cm}}^{2}/\mathrm{mg}$ and pc/μm, and the conversion relationship is shown in Equation (2):

(2) $$1_{}\mathrm{pc}/\mu m=\frac{1\times 10^{-12}C}{1.6\times 10^{-19}C/\mathrm{pair}}\cdot \frac{3.6\mathrm{eV}/\mathrm{pair}}{\rho \times 10^{6}}\cdot 10^{4}=96.608_{}\;\mathrm{MeV}\cdot {\mathrm{cm}}^{2}/\mathrm{mg}$$

The density of silicon is 2.33 g/cm^3^, and the ions produce one electron-hole pair for every 3.6 eV of energy lost on the silicon substrate [11]. The electron-hole pair produced by a single particle incidence can be expressed by Equation (3):

(3) $$\frac{dE}{dx}=\frac{dP}{dx}=\frac{\rho }{3.6\mathrm{eV}/\mathrm{pair}}\cdot LET$$

**Figure 6:**
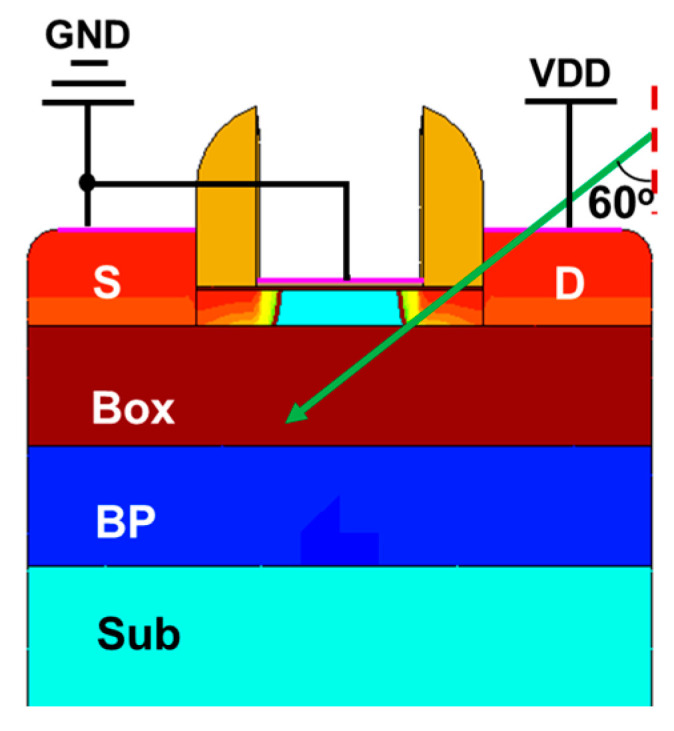
The worst condition of single particle incidence.

**Figure 7:**
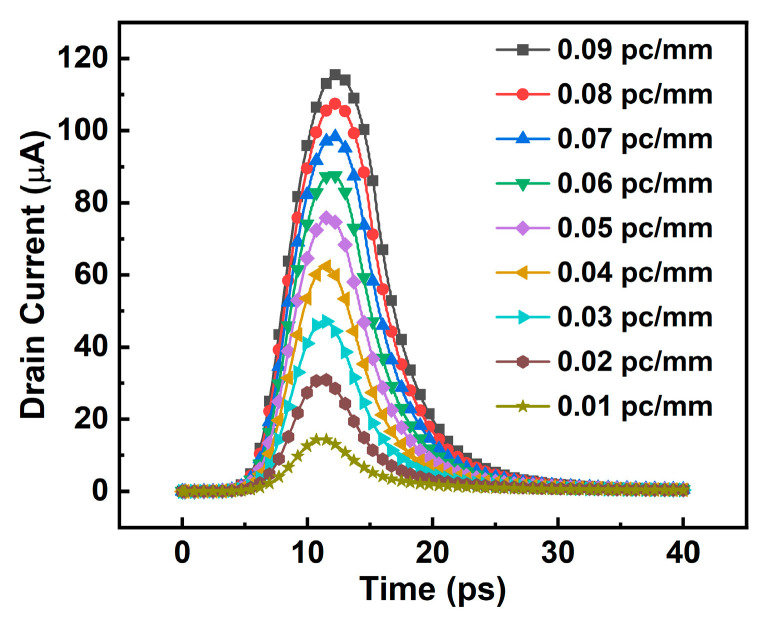
SET currents with different LET values under the worst-case condition.

It is shown that the peak value of the SET current generated by the single particle at the position of high field strength and long particle trajectory is the largest [8,12]. As shown in Figure 6, when the incident position is selected as the drain to channel junction, the incident angle is 60° oblique incidence and the device is in an off-state state; with an incident radius of 15 nm, the obtained SET current is the worst case. The incident radius corresponds to the scattering radius within the device. Under this condition, the SET current under a different *LET* is simulated, and the results are shown in Figure 7.

With the increase in LET, the peak current of the SET currents is also increasing, and the pulse width is also larger [13]. There is a positive correlation between the LET and SET currents. This can be attributed to the fact that as the LET increases, heavier ions lose more energy per unit length within the device. Consequently, this results in a higher generation of electron-hole pairs, thereby enhancing the charge collection at the drain and ultimately leading to an improved peak current of SET.

The single particle current is fitted by the Weibull model. The Weibull function has great flexibility in fitting random data, and can well describe the complex transient current waveform caused by SET [14], as expressed in Equation (4).

(4) $$I(t)=a\times {\left(\frac{c-1}{c}\right)}^{\frac{1-c}{c}}\times {\left(\frac{t}{b}\right)}^{c-1}\times e^{-\left({\left(\frac{t}{b}\right)}^{c}-\frac{c-1}{c}\right)}$$

**Figure 8:**
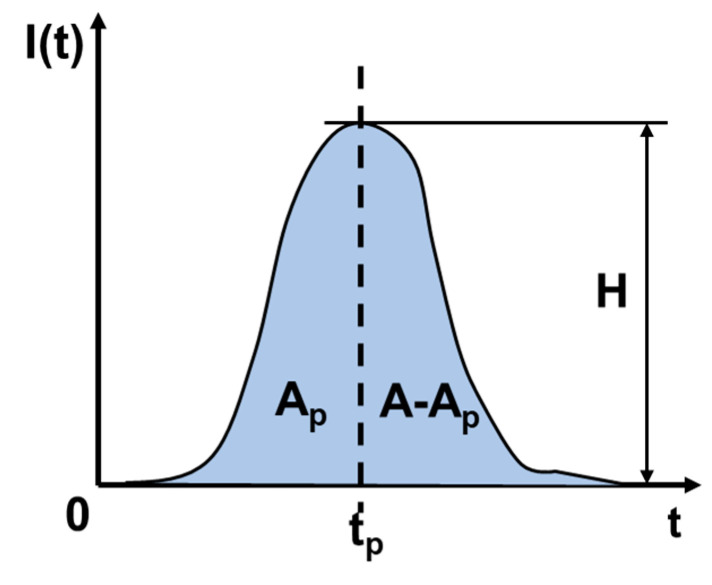
Schematic diagram of SET current.

The three-parameter Weibull model is used, where *t* is the time, and *a*, *b*, *c* are the three parameters introduced. As shown in Figure 8, for any SET current, *H* represents the pulse peak, *t_p_* represents the time when the current pulse reaches the peak, *A* represents the integration of the current pulse and time, and *A_p_* represents the integration of the current pulse in the period of 0~*t_p_*. The Weibull function is described by three important parameters *a*, *b* and *c*, and the expression is shown in Equation (5).

(5) $$\left\{\begin{array}{l} P=\frac{AP}{A}\\ a=H\\ b=\left(\frac{A\cdot c}{a}\right)\cdot {\left(\frac{c-1}{c}\right)}^{\frac{c-1}{c}}\cdot e^{\left(\frac{1-c}{c}\right)}\\ c=\frac{1}{1+\ln\left(1-p\right)} \end{array}\right.$$

### 3.2. 6T-SRAM Single Event Upset

**Figure 9:**
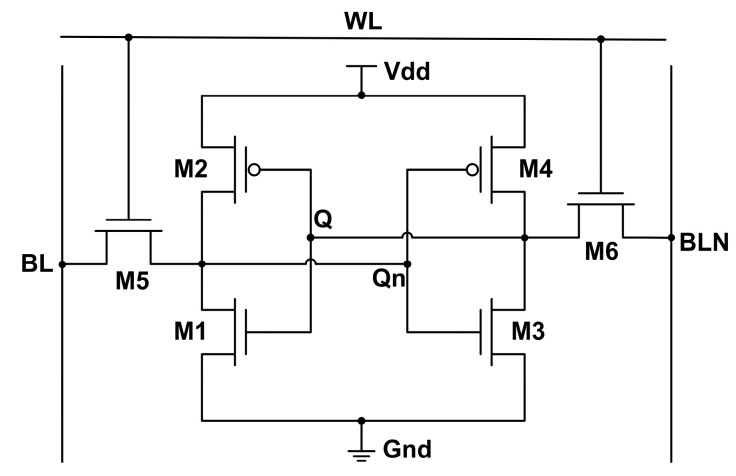
6T-SRAM circuit structure.

The 6T-SRAM circuit is built by a Hspice simulator. As shown in Figure 9, the circuit consists of six transistors, of which M2 and M4 are PFETs, and the remaining transistors are NFETs. The transistor parameter settings are shown in Table 3. The voltage of the circuit node is determined by the word line (WL) and the bit lines (BL, BLN). When the WL is high, M5 and M6 conduct, making the potential of the Q and Q_n_ points change. M1 and M2, M3, and M4 constitute two inverters, respectively. They form a head-to-tail structure to ensure that the potentials of Q and Q_n_ are always opposite. When the Q is high, the Q_n_ is bound to be low, and vice versa.

**Figure 10:**
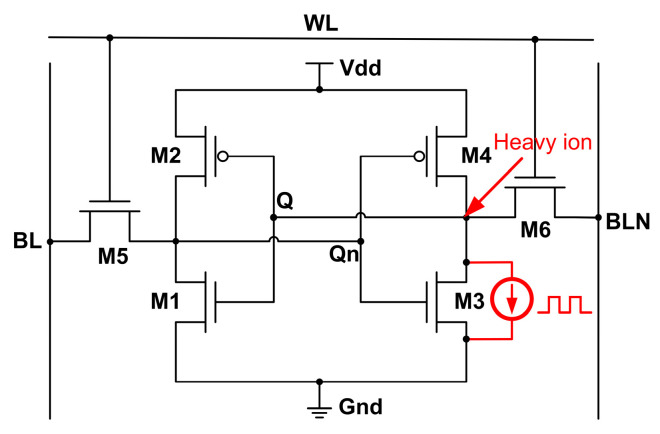
SRAM circuit level simulation.

**Figure 11:**
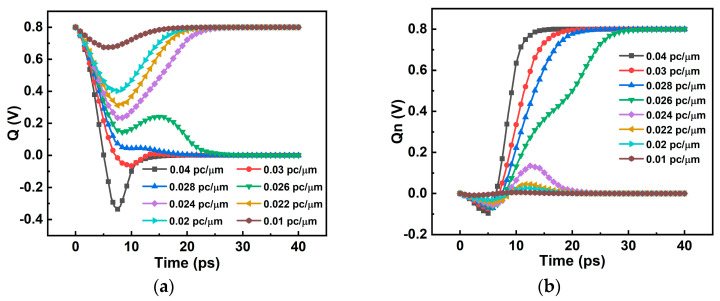
SRAM node voltage variation: (**a**) Q; (**b**) Q_n_.

As shown in Figure 10, Q, as a sensitive node in SRAM circuit, is vulnerable to single event bombardment, resulting in logic flipping of the circuit [15]. M3 is selected as the single event injection device, and the Weibull current source model is added at the Q to simulate the SET current. The variation of the SRAM node voltage is shown in Figure 11.

**Figure 12:**
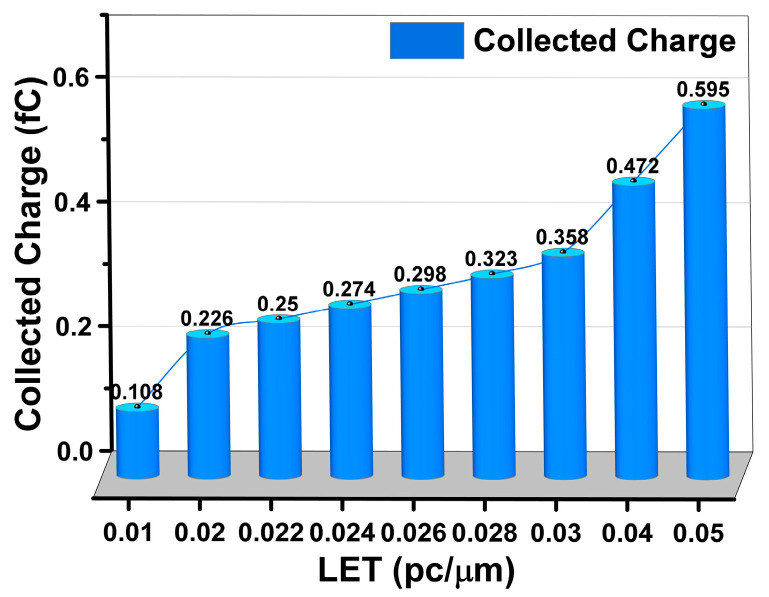
Collected charge for different pulse currents.

When the LET = 0.01 pc/μm SET current is added to the Q node, the voltage of the Q and Q_n_ nodes changes but finally returns to the initial state, and the level logic does not change. The whole process lasts about 25 ns. It shows that the 6T SRAM circuit has the ability to resist SET, and can recover itself to its original state without SEU under the SET current of LET = 0.024 pc/μm. When using a current model with a LET greater than or equal to 0.026 pc/μm, the potentials of the Q and Q_n_ nodes in the SRAM do not return to their initial potentials, and SEU occurs. The current is integrated to obtain the charge collected by the device during the single-particle incident period, which is the critical charge of the device. When the charge collected by the device is greater than the critical charge, the circuit will initiate SEU. Table 4 is the accumulated charge under a different LET value. From the analysis of Figure 11 and Figure 12, it can be seen that the LET upset threshold of the 6T SRAM circuit is in the range of 0.024~0.026 pc/μm, and the critical charge range is 0.274~0.298 fC.

## 4. SRAM Device Level SEE Simulation

**Figure 13:**
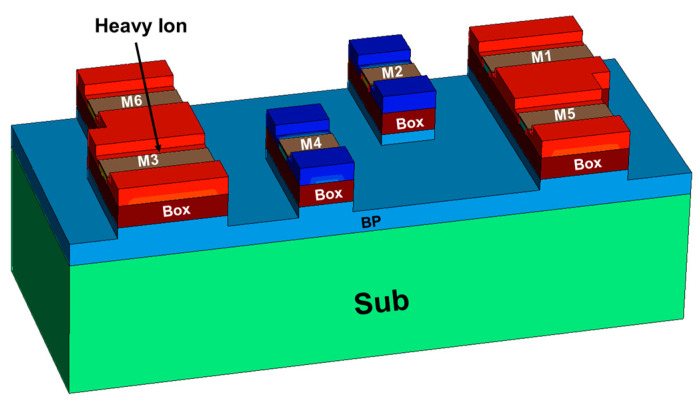
Single event incidence SRAM.

**Figure 14:**
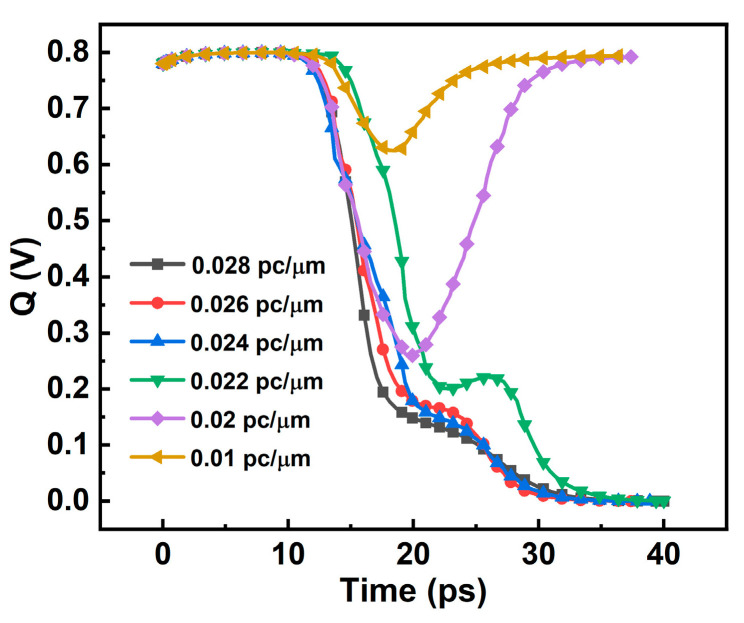
SRAM node voltage variation considering charge sharing effect.

**Figure 15:**
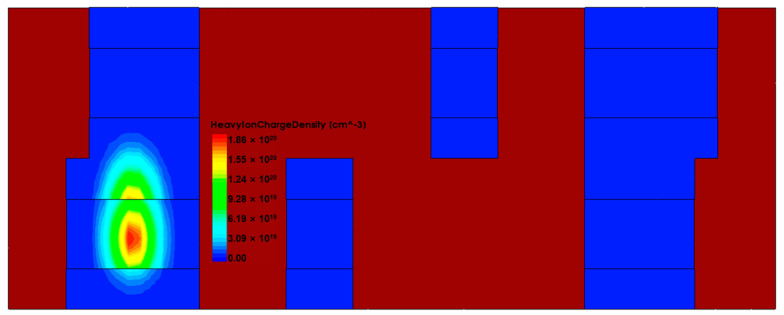
The charge density of heavy ions produced by particle incidence.

SRAM device level simulation uses the same single-event incidence conditions as the circuit level simulation. As shown in Figure 13, the voltage variation of the SRAM node considering the charge sharing case is obtained by performing a 60° oblique incidence at the junction of the drain and channel of the M3 device. The voltage change in the Q node is shown in Figure 14, and the heavy ion charge density is shown in Figure 15.

**Figure 16:**
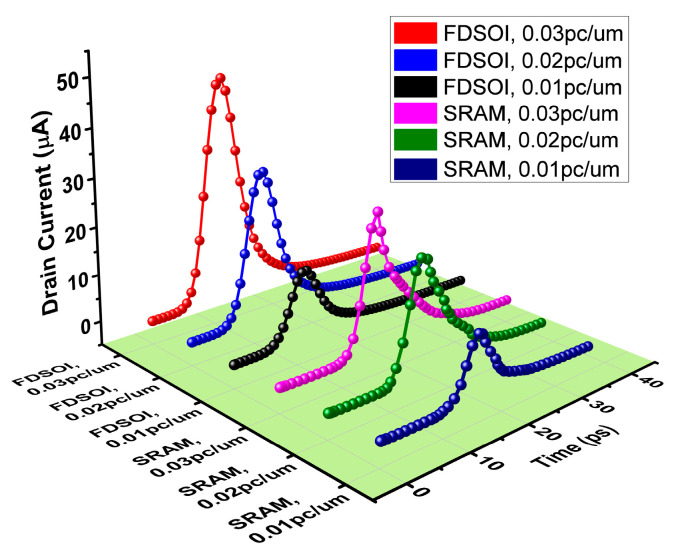
The comparison between the Q node current of SRAM circuit and the SET current of FDSOI.

The results show that the charge sharing effect causes the SRAM circuit to be more prone to SEU. In the SRAM device level simulation, the logic flip occurs when the Q node is incident by a single particle with an LET of 0.022 pc/μm. Comparing the current of the Q node in the SRAM circuit with the SET current of FDSOI, the results are shown in Figure 16. The LET flip threshold is in the range of 0.02~0.022 pc/μm, and the critical charge is in the range of 0.211~0.228 fC. Compared with the circuit level simulation results, the LET flip threshold is reduced by 15.4%, and the critical charge is reduced by 23.5%. The sharing of active regions in an SRAM circuit leads to an increased area of sensitive regions (the drain regions of M1 and M3), indirectly raising the probability of being impacted by a single particle. When a single particle enters M5 (or M6), the electron-hole pairs generated in the drain region of M5 (or M6) will be collected by the drain electrode of M1 (or M3). This phenomenon occurs due to the sharing of active regions in the SRAM layout. Due to the presence of a BOX layer, the impact of electron-hole pairs in the substrate on the transistor is minimal. However, for the top silicon layer, the electrons and holes generated by the incident device can move through the shared active region, enabling charge sharing.

**Figure 17:**
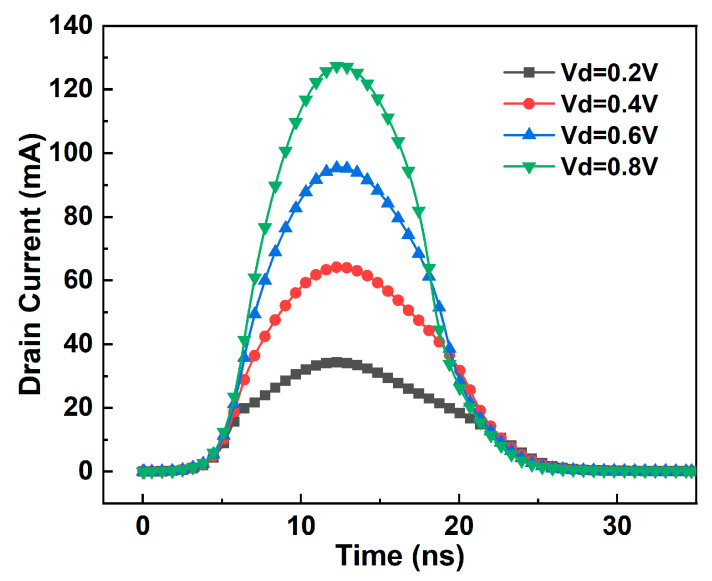
The SET current of FDSOI devices with different drain voltage biases.

At the same LET, the pulse current generated by the single-event incidence of the FDSOI device is greater than the current obtained from the SRAM device-level simulation. This discrepancy is caused by the bipolar amplification effect. In addition to the incident angle, position, and direction of particles, the bias state of the device being irradiated also influences the bipolar amplification effect, thereby indirectly affecting the SET current. Figure 17 presents SET currents generated by heavy ion irradiation in FDSOI under different drain biases. The results demonstrate that varying drain bias voltages result in different peak values of the SET current. During simulations of FDSOI devices, the drain voltage of the device is fixed. In the simulation, the drain voltage of the FDSOI (equivalent to the Q node in SRAM circuit) and the added current model are independent of each other, and the change in the Q point voltage has no effect on the pulse current. But in fact, with the change in the Q point voltage, the SET current also changes. The larger the drain voltage of the device, the larger the SET current, and vice versa.

When an SRAM circuit is in the hold state, assuming the Q node voltage is Vdd, both M6 and M3 are in an off state since the drain of M6 is connected to the drain of M3. When a single particle impacts M6, the ionization generates electrons that drift under the influence of the applied electric field and eventually get collected by the drain (Q). The holes generated within the bulk region, due to their low mobility, partly recombine with electrons in the bulk region, while the remaining portion remains in the bulk region. This causes an increase in the potential of the bulk region, lowering the source/bulk junction barrier and allowing electron injection from the source region into the channel. These electrons can also be collected by the drain, increasing the total amount of charge collected at the drain and resulting in bipolar amplification. This highlights the inadequacy of using non-coupled circuit-level simulations and emphasizes the necessity of employing coupled device-level simulations or hybrid-level simulations to accurately evaluate the circuit’s resistance to SEU. Circuit-level simulations, which do not account for these factors, fail to properly assess the ability of the circuit to withstand SEU.

Due to the shared active region in the SRAM layout, neighboring devices are also influenced when a single particle enters an individual device [16,17]. The sharing of active regions in an SRAM circuit leads to an increased area of sensitive regions (the drain regions of M1 and M3), indirectly raising the probability of being impacted by a single particle. When a single particle enters M5 (or M6), the electron-hole pairs generated in the drain region of M5 (or M6) will be collected by the drain electrode of M1 (or M3). This phenomenon occurs due to the sharing of active regions in the SRAM layout. Due to the presence of a BOX layer, the impact of electron-hole pairs in the substrate on the transistor is minimal. However, for the top silicon layer, the electrons and holes generated by the incident device can move through the shared active region, enabling charge sharing. Furthermore, experimental results [18] also demonstrate the existence of charge-sharing effects in FDSOI SRAM. This indicates that the decrease in the LET upset threshold observed in device level simulations of SRAM is a result of the combined effects of charge sharing and bipolar amplification.

**Figure 18:**
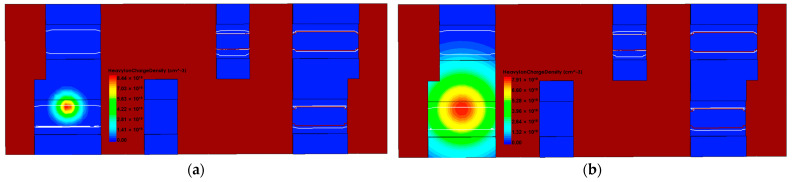
Heavy ion charge density at different radii of incidences: (**a**) 15 nm; (**b**) 50 nm.

**Figure 19:**
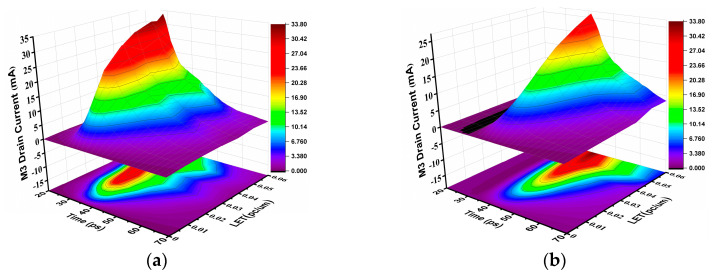
SET current at different incidence radii: (**a**) 15 nm; (**b**) 50 nm.

**Figure 20:**
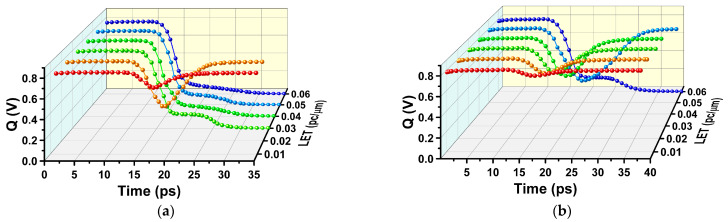
Q node voltage variation of SRAM circuits with different incidence radii: (**a**) 15 nm; (**b**) 50 nm.

To study the effect of the trajectory radius of particle incidence on the SEU of SRAM, heavy ion with radii of 15 nm and 50 nm are used for incidence. The incidence position is the junction of the M3 device drain and channel, the incidence direction is vertical incidence, and the heavy ion charge density is shown in Figure 18, and the Q node voltage and current of SRAM are shown in Figure 19 and Figure 20.

At the same incident energy, the incident particles with a small radius can cause worse results. Because the particle radius is smaller, the generated heavy ion charge density is larger, and the peak of the SET current will be larger, resulting in the SRAM circuit being more prone to logic flip.

## 5. Conclusions

In this paper, the circuit level and device level simulation of 6T-SRAM SEU are compared. It is found that the SEU threshold of the SRAM circuit obtained by the Hspice simulator at the circuit level is larger than the device level simulation. This is due to the combined effect of charge sharing and bipolar amplification, resulting in both the irradiated device and its neighboring devices being influenced by the SET current. This effect is reflected in the circuit as multiple nodes of the SRAM experiencing simultaneous current pulses, making it more prone to logical flips. The LET flip threshold of a 6T-SRAM device level simulation is reduced by 15.4% and the critical charge is reduced by 23.5% compared to the circuit level simulation. Comparing the effects of different particle incident radii on the SEU of SRAM, the results show that particles with smaller incident radii at the same incident energy are more likely to cause SEU. It shows that the energy of the particles is not the only determinant of the circuit logic flip [19], and the ionization distribution generated by the particle incident has a great influence on the electrical characteristics of the device.

## Figures and Tables

**Table 1:** Device parameters used for simulation of NFET and PFET devices.

Parameter	NFET	PFET
Gate length (L_g_, nm)	22	22
Gate Width (W_NW_, nm)	92	46
Channel thickness (T_si_, nm)	6	6
Gate oxide thickness (T_ox_, nm)	1.5	1.5
Buried oxide thickness (T_box_, nm)	20	20
Source/Drain Length (L_s_/L_d_, nm)	38	38
Channel doping (N_ch_, cm^−3^)	10^15^ (acceptor)	10^17^ (donor)
Source/Drain Doping (N_S_/N_D_, cm^−3^)	10^20^ (donor)	10^20^ (acceptor)
Gate work function (*φ_m_*, eV)	4.47	4.72

**Table 2:** DC parameters of the devices.

Parameter	NFET	PFET
Sub-threshold slope (SS, mV/dec)	81.70	81.882
Maximum transconductance (g_m_, _max_, μS)	186.40	65.26
On-state Current (I_on_, μA)	74.38	−22.27
Off-state Current (I_off_, pA)	125.10	−22.48

**Table 3:** 6T-SRAM circuit parameters.

Device	W	L	W/L
M1	92	22	4.18
M2	46	22	2.09
M3	92	22	4.18
M4	46	22	2.09
M5	76	22	3.45
M6	76	22	3.45

**Table 4:** Collected charge for different pulse currents.

LET (pc/μm)	Collecting Charge (fC)
0.01	0.108
0.02	0.226
0.022	0.250
0.024	0.274
0.026	0.298
0.028	0.323
0.03	0.358
0.04	0.472

## Data Availability

Not applicable.
